# Mobile phone dependence and musculoskeletal pain prevalence in adolescents: a cross-sectional study

**DOI:** 10.3389/fpain.2025.1489293

**Published:** 2025-03-19

**Authors:** David Mauricio Parra-Fernandez, Margareth Lorena Alfonso-Mora, María Alejandra Sánchez-Vera, Paola Sarmiento-Gonzalez, Andrea Milena García Becerra, Miriam Guerra-Balic

**Affiliations:** ^1^ Physiotherapy, Universidad de La Sabana, Chía, Colombia; ^2^ Nursing, Universidad de La Sabana, Chía, Colombia; ^3^ Faculty of Psychology and Behavioral Sciences, Universidad de La Sabana, Chía, Colombia; ^4^ Facultat de Psicologia, Ciències de l'Educació i de l'Esport Blanquerna, Universitat Ramon Llull, Barcelona, Catalonia, Spain

**Keywords:** musculoskeletal pain, mobile phone use, adolescent, physiotherapy, low back pain, neck pain

## Abstract

**Objective:**

To investigate the association between adolescents' mobile phone dependence (MPD) and musculoskeletal pain.

**Methods:**

A cross-sectional study was conducted among 622 adolescents aged 10–18 in Tabio, Colombia. Participants completed an online survey that included the MPD and the Nordic Musculoskeletal Questionnaire, which assessed musculoskeletal pain symptoms.

**Results:**

56.3% (*n* = 350) participants reported experiencing musculoskeletal pain, with the upper back being the most affected area (30.4%, *n* = 193). Adolescents reporting pain had significantly higher MPD scores compared to those without pain (mean 29 vs. 24, *p* < 0.001). Additionally, females exhibited higher MPD scores than males (mean 29 vs. 25, *p* < 0.001) and had a higher prevalence of pain (32% vs. 24%). Furthermore, older adolescents in the 11th grade had higher MPD scores than younger adolescents in the 5th grade (mean 31 vs. 21, *p* < 0.019). Logistic regression analysis indicated that specific MPD dimensions, namely “abuse” and “difficulty regulating use,” were significantly associated with general pain and neck pain, but no association was observed with upper back pain. Furthermore, female sex was linked to both neck and upper back pain.

**Conclusion:**

This study found that the MPD dimensions of “abuse” and “difficulty regulating use” were significantly associated with neck pain, regardless of the adolescents' sex.

## Introduction

The widespread adoption of mobile phones has resulted in significantly increased screen time and sedentary behaviors among adolescents (1, 2). This trend raises concerns about the potential burden on the musculoskeletal system. Globally, mobile phone usage is growing, with 5.44 billion users representing 68% of the world's population (1). Among adolescents, usage has dramatically increased, with a 17% rise observed between 2019 and 2021 (2), with approximately 70% of Latino youth owning a mobile phone. Adolescents frequently use these devices for social media, video streaming, and gaming, with a weekly average usage increasing from 8 to 10 h (2). In Colombia, the prevalence of mobile phone ownership among adolescents is approximately 61.7%, with 30.6% reporting daily use ranging from 30 min to 1 h (3).

While mobile phones offer adolescents numerous benefits, including increased autonomy, improved academic performance, and enhanced social interaction (4, 5), excessive use is associated with considerable risks to physical, mental, and psychosocial well-being (6, 7). Problematic mobile phone use is particularly prevalent among 15- to 16-year-olds, as it is considered the most common way of dependency disorder during adolescence (8–12). This is characterized by excessive and frequent use (13), academic disruption, and emotional distress (14).

Emerging evidence strongly suggests a link between problematic mobile phone use and musculoskeletal disorders (MSKDs) in adolescents (15–18). MSKDs include a range of conditions affecting bones, muscles, joints, and connective tissues. Representing a major contributor to the global disease burden in individuals aged 15–65, MSKDs affect an estimated 1.7 billion people worldwide (19). The prevalence of MSKDs in adolescents is estimated at 47.4%, with pain being the primary reported symptom (20). Studies indicate a higher incidence of MSKDs associated with excessive mobile phone use among females compared to males (18, 20), particularly among those aged 15–17 years (21).

The most reported pain sites include the neck, shoulders, wrists/hands, upper back, and lower back; some adolescents also report vision-related symptoms (7, 8, 15–17, 20–23). Prolonged mobile phone use (more than 10 h per week) significantly increases the risk of neck and lower back pain. Furthermore, lifestyle factors such as physical inactivity, poor sleep, inadequate fruit and vegetable consumption, and substance use have been identified as predictors of persistent MSKDs (24), in addition to the posture adopted during mobile phone use (25).

Given these concerns, this study investigated the association between MPD and MSKDs in adolescents residents of the Sabana Centro region of Colombia. It is the first to assess the prevalence of pain associated with problematic mobile phone use within the Colombian population. Its relevance is heightened by the substantial increase in screen time following the COVID-19 pandemic, impacting both educational and non-educational settings. A comprehensive understanding of problematic screen use, particularly concerning mobile phones, is crucial for developing effective interventions to improve adolescents' health and well-being.

## Methods

### Study design

A cross-sectional design was applied to investigate the association between MPD and musculoskeletal pain among adolescents. Data were collected from two educational institutions in Tabio, Colombia, during September and October 2022. Ethical approval was received by the ethics committee of the University of La Sabana (Minute 021-18 Nov 2020). Before participation, all students provided informed assent, and their parents/guardians signed informed consent forms.

### Sample size calculation

The sample size was calculated using R Studio 4.3.2.1 and the pwr package. A binomial statistical test was employed, assuming a small effect size [Cohen's *h* = 0.03 (8)], with a significance level (*α*) set to 0.05 and a power of 0.80. Based on these parameters, the required sample size was 622 participants (11).

### Participants

For the recruitment process, 1,183 students from the two schools were invited to volunteer after receiving information about the study's objectives. A researcher administered the survey in their classrooms, and consent forms were sent to parents to obtain their signed approval. Participants were included if they were school-aged (10–18 years), enrolled in grades 5–11th, and had access to a mobile phone. Exclusion criteria were a pre-existing medical diagnosis of a disability. Of the 1,183 invited students, 685 consented to participate; after applying inclusion/exclusion criteria, 63 participants were excluded, resulting in a final sample of 622 adolescents.

### Variables and instruments

Participants completed an online questionnaire comprising three sections:

1. Sociodemographic data: age, sex, and grade level.
2. Mobile phone dependence MPD: the MPD scale was used to assess MPD. This scale, designed for adolescents consisted of 22 items grouped into four factors: withdrawal (36 points), abuse and difficulty (36 points), problems arising from use (16 points), and tolerance/interference (16 points). Items are rated on a 5-point Likert scale (0 = never to 4 = always), with a total score ranging from 0 to 88. Higher scores reflect greater dependency. The validated Spanish version of the MPD scale (Cronbach's alpha = 0.89) was used (5).
3. Musculoskeletal pain assessment: musculoskeletal pain experienced in the previous 6 months was assessed using a Spanish-validated version of the Nordic Musculoskeletal Questionnaire (NMQ) (26). The reliability of this version demonstrated an intraclass correlation coefficient (ICC) exceeding 0.9. Participants responded “yes” or “no” to five questions regarding pain in the following five specific body regions: (1) neck, (2) head and neck, (3) shoulders, (4) upper back, and (5) lower back. Each question included a visual diagram of the corresponding body region. General pain was defined as pain in any of these five regions.

### Statistical analysis

Data analysis was done using SPSS version 26. Descriptive statistics (frequencies, percentages, means, median standard deviations, interquartile ranges) were obtained. The Shapiro–Wilk test was applied to assess the normality of data distribution. Given the non-normal distribution of mobile phone dependency scores, non-parametric tests (Spearman correlation for correlations, Mann–Whitney *U* test for group comparisons) were used. A Spearman correlation coefficient (*ρ*) of 0.76–1.00 was considered a strong relationship (27). The *χ*^2^ test was used to identify the association between sex and pain prevalence. A *p*-value ≤0.05 was considered statistically significant. Logistic regression analysis was used to model the relationship between general pain, neck pain, and upper back pain (dichotomous outcome: yes/no), with the independent variables of sex, age, and MPD factors.

## Results

Of the 1,183 students invited, 622 (52%) participated in the study, including pupils from grades 5–11th, of which 54.5% were females and 45.5% were males, with a median age of 14 years. Musculoskeletal pain (in at least one of five specified body regions) was reported by 56.3% (*n* = 350) participants within the past 6 months. The upper back was the most frequently affected region (30.4%, *n* = 193), followed by the neck (29.3%) and shoulders (21.1%). The median MPD score was 27 [interquartile range (IQR): 17–37] (Table 1).

**Table 1:** Sociodemographic characteristics and prevalence of musculoskeletal pain.

Variable	Category	*n*	%
Sex	Male	283	45.5%
	Female	339	54.5%
Grade	Fifth	58	9.3%
	Sixth	99	15.9%
	Seventh	72	11.6%
	Eighth	88	14.1%
	Ninth	116	18.6%
	Tenth	116	18.6%
	Eleventh	73	11.7%
General pain	Yes	350	56.3%
	No	272	43.7%
Neck pain	Yes	186	29.9%
	No	436	70.1%
Head & neck pain	Yes	117	18.8%
	No	505	81.2%
Shoulder pain	Yes	134	21.5%
	No	488	78.5%
Upper back pain	Yes	193	30.4%
	No	429	69.6%
Lower back pain	Yes	99	15.9%
	No	523	84.1%
Age	14 (12–16) Median (IQR)		
MPD Score	27.0 (17–37) Median (IQR)		

Qualitative data is presented as frequency and percentage, numerical data is presented as medians and interquartile range (IQR), general pain is the presence of pain in any region of the back and shoulder; MPD, test of mobile phone dependence.

Female adolescents reported significantly higher MPD scores than males in all dimensions except for Excessive Use (median difference in total MPD: 4 points; *p* < 0.001). MPD scores increased significantly with the grade level (*p* = 0.019). Participants reporting pain in any body region exhibited significantly higher MPD scores (median difference: 5 points; *p* < 0.001) compared to those without pain, except for those with low back pain (*p* = 0.055) (Table 2).

**Table 2:** MPD scores by demographic factors and pain status.

Variable	Category	MPD score median (IQR)	*p*-value
Sex	Male	25 (24.1–27.2)	<0.001**
	Female	29 (28.2–30.0)	
Grade	Fifth	21.5 (19.8–28.0)	<0.019*
	Sixth	22.0 (21.7–26.5)	
	Seventh	24.5 (23.2–29.6)	
	Eighth	26.0 (24.9–31.5)	
	Ninth	27.5 (25.6–31.2)	
	Tenth	29.0 (27.5–32.6)	
	Eleventh	31.0 (30.4–37.1)	
Pain status	General pain: no pain	24.0 (23.4–26.8)	<0.001**
	General pain: pain	29.0 (28.7–31.8)	
	Neck pain: no pain	25.0 (24.8–27.5)	<0.001**
	Neck pain: pain	31.0 (31.1–34.6)	
	Head & neck pain: no pain	26.0 (24.6–31.2)	0.035**
	Head & neck pain: pain	32.0 (32.3–37.7)	
	Shoulder pain: no pain	30.0 (28.7–33.6)	0.012**
	Shoulder pain: pain	32.0 (30.8–39.2)	
	Upper back pain: no pain	30.0 (27.9–33.4)	0.007**
	Upper back pain: Pain	31.0 (30.9–37.5)	
	Lower back pain: no pain	30.0 (28.7–33.6)	0.055
	Lower back pain: pain	31.0 (31.6–40.2)	

* *p* value derived from Kruskal Wallis Test.

** *p* value derived from Mann‒Whitney test.

The analysis of MPD dimensions by gender revealed consistently higher female scores across all dimensions except “problems arising from use” (Table 3). Among participants with back pain, those experiencing pain in the neck, shoulders, or upper back had significantly higher scores on three of the four MPD dimensions; however, no significant difference was observed for low back pain (Table 4).

**Table 3:** MPD dimension scores by sex.

MPD dimension	Sex	MPD score median (IQR)	*p*-value
Withdrawal	Male	3 (3.6–4.6)	<0.005**
	Female	4 (4.8–5.8)	
Abuse/difficulty	Male	14 (13.1–14.6)	<0.001**
	Female	16 (15.8–16.6)	
Excessive use	Male	2 (2.2–2.8)	0.452
	Female	2 (2.2–2.7)	
Tolerance	Male	5 (4.6–5.4)	<0.001**
	Female	6 (5.8–6.5)	

** *p*-value < 0.01.

**Table 4:** The median difference between mobile phone dependence factors and pain.

MPDF	GP		NP		HNP		SP		UBP		LBP	
	No	Yes	No	Yes	No	Yes	No	Yes	No	Yes	No	Yes
Total score MPD	24 (23.4–26.8)	29 (28.7–31.7)*	25 (24.8–27.5)	25 (28.7–31.7)*	26 (24.4–30.9)	32 (32.2–37.6)*	26 (25.6–28.1)	29.5 (29.5–34.9)*	26 (25.7–27.7)	30 (29.4–33.7)*	26 (26.1–28.6)	29 (27.8–33.9)
Withdrawal	3.0 (3.7–4.8)	4.0 (4.7)*	3 (4–4.9)	5 (4.9–6.3)*	3 (2.9–4.7)	6 (5.8–7.6)*	3 (4.0–4.8)	5 (5.1–6.9)*	3 (4.0–4.8)	5 (4.9–6.3)*	3 (4.2–5.0)	4 (4.5–6.5)
Abuse and difficulty	13.0 (12–14)	16.0 (15.4–16.8)*	14 (13.3–14.6)	17.0 (16.1–18.2)*	15.5 (13.6–16.8)	18 (17.0–19.6)*	14 (13.9–15.1)	16 (15.3–17.7)*	14 (13.5–14.8)	16 (15.6–17.5)*	14 (14.0–15.2)	15 (15.0–17.9)*
Excessive use	2 (2.0–2.6)	2 (2.3–2.9)	2 (2.1–2.5)	2 (2.5–3.2)*	2 (2.3–3.5)	2 (2.3–3.3)	2 (2.2–2.6)	2 (2.5–3.3)*	2 (2.1–2.6)	2 (2.4–3.2)	2 (2.3–2.7)	2 (2.0–3.1)
Tolerance	5 (4.6–5.4)	6.0 (5.8–6.5)*	5 (5–5.6)	6 (6.0–7.0)*	5 (4.9–6.5)	7 (6.3–7.5)*	5 (5.1–5.7)	6 (6.0–7.9)*	5 (5.0–5.6)	6 (6.0–7.0)*	5 (5.2–5.8)	6 (5.6–7)

GP, general pain; NP, neck pain; HNP, head and neck pain; SP, shoulder pain; UPB, upper back pain; LBP, low back pain; MPDF, mobile phone dependence factors.

* Significant differences *U* the Mann–Whitney *p* value <0.05 data are presented in median and interquartile range.

A weak positive correlation was found between age and total MPD scores (Rho = 0.19, *p* < 0.01). This association was particularly pronounced for the “withdrawal” (Rho = 0.15, *p* < 0.01), “abuse/difficulty” (Rho *ρ* = 0.16, *p* < 0.01), and “tolerance” (Rho = 0.25, *p* < 0.01) dimensions of the MPD scale. Females reported a significantly higher prevalence of pain (approximately 10% greater than males) across most body regions, except for the low back and shoulders (*χ^2^* test, *p* < 0.001). Furthermore, adolescents reporting pain were significantly older (mean difference of 1 year, *p* < 0.001) than those without pain (Table 5).

**Table 5:** Prevalence of musculoskeletal pain by sex and age.

Pain location	Sex	Pain prevalence (%)	*p*-value (Sex)^+^	Pain	Age (years)—median (IQR)	*p*-value (Age)^++^
General pain	Male	23.5%	<0.001**	Pain	14 (13.2–13.7)	<0.001**
	Female	**32.8**%		No pain	15 (14.3–14.7)	
Neck pain	Male	11.0%	<0.003**	Pain	15 (13.6–14.0)	<0.001**
	Female	**19.0**%		No pain	15 (14.3–14.8)	
Head & neck pain	Male	18.4%	0.008*	Pain	14 (13.7–14.6)	0.019*
	Female	**44.9**%		No pain	15 (14.5–15.2)	
Shoulder pain	Male	8.1%	0.034*	Pain	14 (13.7–14.1)	0.012*
	Female	13.5%		No pain	14.5 (14.1–14.7)	
Upper back pain	Male	11.8%	0.010*	Pain	14 (13.5–13.9)	<0.001**
	Female	**19.4**%		No pain	15 (14.4–15.0)	
Lower back pain	Male	5.5%	0.015*	Pain	14 (13.7–14.1)	<0.001**
	Female	**10.5**%		No pain	15 (14.2–15.0)	

IQR, interquartile range.

A bold values means the highest frequency of pain by sex.

^+^ *p* value derived from the chi-square test.

^++^ *p* value derived from the *U* de Mann–Whitney test.

* *p*-value < 0.05.

** *p*-value < 0.01.

Three logistic regression models were developed to examine the association between general pain, neck pain, and upper back pain, as these were the most prevalent pain areas reported among the participants. These models included sex, age, and the four dimensions of the MPD scale (withdrawal, abuse/difficulty, problems arising from use, and tolerance/interference) as independent variables. Significant associations were found between the “abuse/difficulty” dimension of the MPD and general and neck pain. Furthermore, female sex was identified as associated variable for neck and upper back pain (Appendix 1).

## Discussion

This study revealed a high prevalence of musculoskeletal pain among adolescents, particularly affecting the upper back and neck. Higher MPD scores were significantly associated with pain and were more prevalent among female and older adolescents. A positive correlation was found between age and MPD scores and between sex and pain prevalence.

The observed prevalence of musculoskeletal pain aligns with previous research. Martins et al. (20) reported a 47.4% prevalence in a similar adolescent population, consistent with our findings of 56.3%. Straker et al. (18) found neck and shoulder pain in 50% of participants, with lower back pain in 34% and upper limb pain in 30%, supporting the high prevalence of upper body pain found in our study. Mustafaoğlu et al. (22) also reported high prevalence rates of upper back, neck, and wrist/hand pain, and similar observations regarding neck, shoulder, and eye discomfort have been reported by Ahmed et al. (17) and Mokhtarinia et al. (8).

Previous studies on mobile phone dependency have shown varying prevalence rates. Mokhtarinia et al. (8) reported a 53.3% prevalence in Iran, with slightly higher rates among males (54.5%) than females (52.7%), although no significant difference in average dependency scores was found. Gangadharan et al. (7) reported a lower prevalence (33%) in India, with slightly higher rates among males (33.6%) than females (32.3%). Alsalameh et al. (15) also observed higher rates in males. In contrast, our findings are consistent with Straker et al. (18), who reported higher mobile phone use among older adolescents and females.

The observed discrepancies regarding sex and MPD between our study and prior research might be attributable to several factors. Our study included a wider age range, which may have influenced the results. Furthermore, our larger sample size allowed for greater statistical power to detect significant differences. The observed higher mobile phone dependency among females supports further investigation into potential gender-specific patterns of mobile phone usage.

Our findings showed a significant association between higher MPD scores and musculoskeletal pain, primarily in the neck, shoulders, and upper back, but not in the lower back. This is consistent with Alsalameh et al. (15), who also found higher MPD scores among participants with pain (except those with elbow or lower back pain). However, our study only revealed a significant correlation between MPD and age. The gender differences in pain prevalence align with Sirajudeen et al. (28) who observed an association between sex and shoulder pain, although Walankar et al. (29) did not report a similar relationship. These variations may reflect differences in study populations, cultural contexts, or methodological approaches.

This cross-sectional study has several limitations. Its design does not allow for establishing causality between MPD and musculoskeletal pain. Additionally, other forms of screen time potentially associated with pain, such as the use of computers and tablets, were not assessed. Self-reported pain data are subject to bias, which could lead to underreporting or overreporting of results. Finally, selection bias should be considered, as the study included only pupils who returned signed informed assents and whose parents or legal guardians provided signed consent.

Extensive literature links risk mobile phone use to various negative health outcomes, affecting both physical and mental health. The rising prevalence of musculoskeletal pain in adolescents is likely multifactorial, involving excessive mobile device use, sedentary behavior, poor posture (e.g., forward head posture), and sleep disturbances. Muscular fatigue, chronic pain, and reduced quality of life are common consequences (30). Based on this evidence, interventions promoting increased physical activity, sports participation, and limiting screen time (to no more than 2 h daily) are strongly recommended (30).

## Conclusion

The prevalence of musculoskeletal pain is a significant contributor to the global burden of disease, which is increasing among children and adolescents. Lifestyle habits are a risk factor for these conditions in this population. This study found not only a prevalence of 58% for pain but also an association with mobile dependency, particularly among females, with the incidence increasing with age. These findings emphasize the need for public health interventions aimed at adolescents to promote healthier mobile phone use, encourage physical activity, and improve posture, particularly among females. Future research should assess the effectiveness of these interventions, with a specific focus on addressing the “abuse” and “difficulty regulating use” dimensions of MPD.

## Data Availability

The raw data supporting the conclusions of this article will be made available by the authors, without undue reservation.
