# Supplementary material for: Mobile phone dependence and musculoskeletal pain prevalence in adolescents: a cross-sectional study
Source: Front Pain Res (Lausanne). 2025 Mar 19;6:1489293. doi: 10.3389/fpain.2025.1489293 (PMC11961645; doi:10.3389/fpain.2025.1489293)
Supplement: Supplementary file 1 [file Table1.docx]

| General Pain | | | | | | |
| --- | --- | --- | --- | --- | --- | --- |
|  | | β | Standard error | Wald Test | df | Sig. |
| Step 1^a^ | Sex | .272 | .174 | 2.438 | 1 | .118 |
|  | Age | .116 | .099 | 1.360 | 1 | .244 |
|  | Grade | .156 | .106 | 2.162 | 1 | .141 |
|  | Withdrawal | -.035 | .026 | 1.759 | 1 | .185 |
|  | Abuse and Difficulty | .052 | .019 | 7.794 | 1 | .005 |
|  | Excessive Use | -.011 | .040 | .070 | 1 | .792 |
|  | Tolerance | .028 | .038 | .522 | 1 | .470 |
|  | Constante | -2.736 | 1.128 | 5.887 | 1 | .015 |

df: degrees of freedom

| **Neck Pain** | | | | | | |
| --- | --- | --- | --- | --- | --- | --- |
|  | | β | Standard error | Wald Test | df | Sig. |
| Step 1^a^ | Sex | .440 | .190 | 5.378 | 1 | .020 |
|  | Age | .080 | .107 | .566 | 1 | .452 |
|  | Grade | .127 | .114 | 1.245 | 1 | .265 |
|  | Withdrawal | -.023 | .028 | .718 | 1 | .397 |
|  | Abuse and Difficulty | .061 | .020 | 9.605 | 1 | .002 |
|  | Excessive Use | .020 | .041 | .238 | 1 | .625 |
|  | Tolerance | .000 | .041 | .000 | 1 | .994 |
|  | Constante | -3.554 | 1.221 | 8.469 | 1 | .004 |

df: degrees of freedom

| **Upper Back pain** | | | | | | |
| --- | --- | --- | --- | --- | --- | --- |
|  | | β | Standard error | Wald Test | df | Sig. |
| Step 1^a^ | Sex | .378 | .188 | 4.028 | 1 | .045 |
|  | Age | .039 | .107 | .137 | 1 | .711 |
|  | Grade | .253 | .114 | 4.898 | 1 | .027 |
|  | Withdrawal | -.001 | .028 | .001 | 1 | .974 |
|  | Abuse and Difficulty | .025 | .019 | 1.627 | 1 | .202 |
|  | Excessive Use | .023 | .041 | .309 | 1 | .579 |
|  | Tolerance | .018 | .041 | .198 | 1 | .656 |
|  | Constante | -2.979 | 1.216 | 6.006 | 1 | .014 |

df: degrees of freedom
